# Temporomandibular Joint Disorder Complaints in Tinnitus: Further Hints for a Putative Tinnitus Subtype

**DOI:** 10.1371/journal.pone.0038887

**Published:** 2012-06-19

**Authors:** Veronika Vielsmeier, Jürgen Strutz, Tobias Kleinjung, Martin Schecklmann, Peter Michael Kreuzer, Michael Landgrebe, Berthold Langguth

**Affiliations:** 1 Department of Otorhinolaryngology, University of Regensburg, Regensburg, Germany; 2 Department of Otorhinolaryngology, University of Zurich, Zurich, Switzerland; 3 Department of Psychiatry and Psychotherapy, University of Regensburg, Regensburg, Germany; Marienhospital Herne - University of Bochum, Germany

## Abstract

**Objective:**

Tinnitus is considered to be highly heterogeneous with respect to its etiology, its comorbidities and the response to specific interventions. Subtyping is recommended, but it remains to be determined which criteria are useful, since it has not yet been clearly demonstrated whether and to which extent etiologic factors, comorbid states and interventional response are related to each other and are thus applicable for subtyping tinnitus. Analyzing the Tinnitus Research Initiative Database we differentiated patients according to presence or absence of comorbid temporomandibular joint (TMJ) disorder complaints and compared the two groups with respect to etiologic factors.

**Methods:**

1204 Tinnitus patients from the Tinnitus Research Initiative (TRI) Database with and without subjective TMJ complaints were compared with respect to demographic, tinnitus and audiological characteristics, questionnaires, and numeric ratings. Data were analysed according to a predefined statistical analysis plan.

**Results:**

Tinnitus patients with TMJ complaints (22% of the whole group) were significantly younger, had a lower age at tinnitus onset, and were more frequently female. They could modulate or mask their tinnitus more frequently by somatic maneuvers and by music or sound stimulation. Groups did not significantly differ for tinnitus duration, type of onset (gradual/abrupt), onset related events (whiplash etc.), character (pulsatile or not), hyperacusis, hearing impairment, tinnitus distress, depression, quality of life and subjective ratings (loudness etc.).

**Conclusion:**

Replicating previous work in tinnitus patients with TMJ complaints, classical risk factors for tinnitus like older age and male gender are less relevant in tinnitus patients with TMJ complaints. By demonstrating group differences for modulation of tinnitus by movements and sounds our data further support the notion that tinnitus with TMJ complaints represents a subgroup of tinnitus with clinical features that are highly relevant for specific therapeutic management.

## Introduction

Tinnitus is the perception of sound in the absence of any external sound source and it is considered to be a very heterogeneous condition [1]. A large variety of different etiologic factors can cause tinnitus. On a phenotypic level, tinnitus can be perceived unilaterally, bilaterally or centrally in the head, the perceived sound can be tone-like or noise-like and tinnitus can be accompanied by many comorbidities such as hyperacusis, insomnia, anxiety or depression [2], [3], [4]. In face of such heterogeneity, subtyping of different forms of tinnitus has been proposed as a strategy to facilitate both diagnosis and therapy of tinnitus [5]. In order to be clinically useful the different subtypes should be pathophysiologically different, easily distinguishable and predictive for the outcome of specific interventions. The condition of tinnitus consisting of a “typewriter” like sound may serve as a rare but useful example for a subtype which is caused by vascular-nerve conflict and which has been shown to be responsive to carbamazepine treatment [6]. Successful classification criteria would improve both research and clinical management. Thus there is an important need to identify clinical criteria for useful subtyping of tinnitus patients.

Here we investigated whether comorbid temporomandibular joint complaints may constitute such a discriminating criterion. Since the first description by Costen in 1934 [7] the association of tinnitus with temporomandibular joint (TMJ) dysfunction has been confirmed in many studies [8], [9], [10], [11]. In a recent pilot study we found that tinnitus patients with TMJ problems tend to be younger, more frequently female and to have better hearing function in contrast to those with tinnitus but without TMJ symptoms [12].

Moreover, in many cases tinnitus can be modulated by jaw movements [13], [14]. Actually, an improvement of tinnitus symptoms mediated by a specific therapy of TMJ disorders has been reported [8], [15].

**Table 1:** Assessment instruments.

Assessed characteristics	Assessment instrument
clinical and demographic characteristics	Tinnitus Sample Case History Questionnaire (TSCHQ)(Langguth et al. 2007)
tinnitus handicap	Tinnitus Handicap Inventory (THI) (Newman et al)
tinnitus severity	Tinnitus Questionnaire (TQ) (Goebel and Hiller)
depressive symptoms	Beck Depression Inventory (BDI) (Beck et al. 1961)
quality of life	World Health Organisation Quality of Life Scale (WHOQoL)
tinnitus loudness	Numeric Rating Scale (0–10): Loudness
tinnitus discomfort	Numeric Rating Scale (0–10): Discomfort
Tinnitus annoyance	Numeric Rating Scale (0–10): Annoyance
Tinnitus ignorability	Numeric Rating Scale (0–10): Ignorability
Tinnitus unpleasentness	Numeric Rating Scale (0–10): Unpleasentness

Neuronal pathways, by which the trigeminal afferents can interact with the central auditory system, have been identified in animal studies [16]–[17]. Trigeminal input propagates via the trigeminal ganglia and the trigeminal nucleus to the dorsal cochlear nucleus [18], [19] and can influence activity in central auditory pathways [20], especially in case of cochlear damage [21]. Moreover an interaction of somatosensory stimulation with tinnitus loudness has been reported in people with tinnitus [22] and has been interpreted as a hint for the activation of the non-modality specific extralemniscal pathways in tinnitus [23], [24]. Based on (i) the observation that tinnitus is frequently related with TMJ disorders or neck problems, (ii) the finding that many patients can manipulate their tinnitus by jaw, neck or head movements and (iii) the identification of neuronal pathways mediating somatosensory input to the dorsal cochlear nucleus, the concept of “somatosensoric tinnitus” has been proposed [25]. Since then “somatosensoric tinnitus” has been considered as a potentially useful subtype of tinnitus [26], [27], [28], [29], [30] even if data about an association between the comorbidity “TMJ disorder” and the ability to manipulate the tinnitus by jaw or head movements are scarce [14].

Here, we used the Tinnitus Research Initative Database [31] to compare tinnitus patients with and without self-reported TMJ complaints with respect to their clinical characteristics. Special emphasis was set on differences in the ability to modulate the tinnitus by somatosensoric maneuvers in order to test the association between TMJ comorbidity and somatic modulation claimed by the concept of somatosensoric tinnitus.

## Materials and Methods

The data analysis was based on data of the Tinnitus Research Initiative Database. Data management was conducted according to the Data Handling Plan (TRI-DHP V07, May 9th, 2011). Data analysis was conducted according to the Standard Operating Procedure (TRI-SA V01, May 9^th^, 2011), thereby following a study-specific Statistical Analysis Plan (SAP-004, June 27^th^, 2011) that was written according to the SAP template (TRI-SAP, May 12^th^, 2011). Statistical details can be found below. All documents are to be found under http://database.tinnitusresearch.org/. 1204 patients from the Tinnitus Research Initiative (TRI) Database were investigated. Patients presented between 2005 and 2011 at different tinnitus centers worldwide (Regensburg, Aachen, Germany; Antwerp, Belgium; Volta Redonda, Belo Horizonte, and Porto Alegre, Brazil; Buenos Aires, Argentina). Patients completed the self-measurement questionnaires (see table 1) before their first presentation at the clinic. The diagnosis of tinnitus was confirmed by clinical specialists (medical doctors and/or audiologists with experience in the diagnosis and management of tinnitus). Patients with complete information with respect to the question “Do you suffer from temporomandibular disorder?” (answer: yes or no) in the Tinnitus sample case history questionnaire of the TRI case report form were included. Patients gave written informed consent to record their data in the database and to perform analyses with the data. The project has been approved by the local ethics committee (Ethikkommission der Fakultät für Medizin der Universität Regensburg). There was no overlap with an earlier study [12] as data of this former sample were not included in the data analysis.

Assessment was performed before the first consultation in the tinnitus clinics and included the Tinnitus Sample Case History Questionnaire (TSCHQ), the Tinnitus Handicap Inventory, the Tinnitus Questionnaire, the Beck Depression Inventory, the World Health Organisation Quality of Life Scale (WHOQoL), and several tinnitus numeric rating scales (loudness, discomfort, annoyance, ignorability and unpleasantness) (see table 1).

Among these variables, we were interested in demographic characteristics like age, gender and age at tinnitus onset. Furthermore, we investigated the possible masking of the tinnitus by music or sounds and the ability of modulating the tinnitus by somatic maneuvers. In addition, we analyzed tinnitus duration, pulsatile character, onset related events, the self-reported suffering from hyperacusis, and the mean hearing level (dB HL over all measured frequencies (0.125–8 kHz) of both ears).

If no data were available at the screening visit (first consultation), we used data from the baseline visit of a clinical intervention. If both screening and baseline data were available we used the mean of both time points. For continuous variables (e.g., age) we contrasted both groups (with and without TMJ complaints) with Student t-tests. For categorical variables (e.g., gender), we used χ^2^-tests of independence to investigate differences in the proportion of these variables in both groups. We calculated 23 contrasts; to avoid false positive results we declare only contrasts with a Bonferroni corrected significance threshold of 0.0022 as significant (p = 5%/23 = 0.0022).

## Results

261 patients complained about problems of the TMJ (22%), whereas 943 (78%) reported that they have no symptoms of the temporomandibular joint. Tinnitus patients with TMJ disorders complaints were more frequently female (with TMJ disorder: 54%; without: 33%), significantly younger and had an earlier tinnitus onset compared to those without TMJ disorder. Moreover patients with tinnitus and temporomandibular joint disorder were more frequently able to mask their tinnitus by sounds or music (with TMJ disorder: 85%; without: 74%) and they could more frequently modulate their tinnitus by somatic maneuvers (with TMJ disorder: 48%; without: 30%).

**Figure 1:**
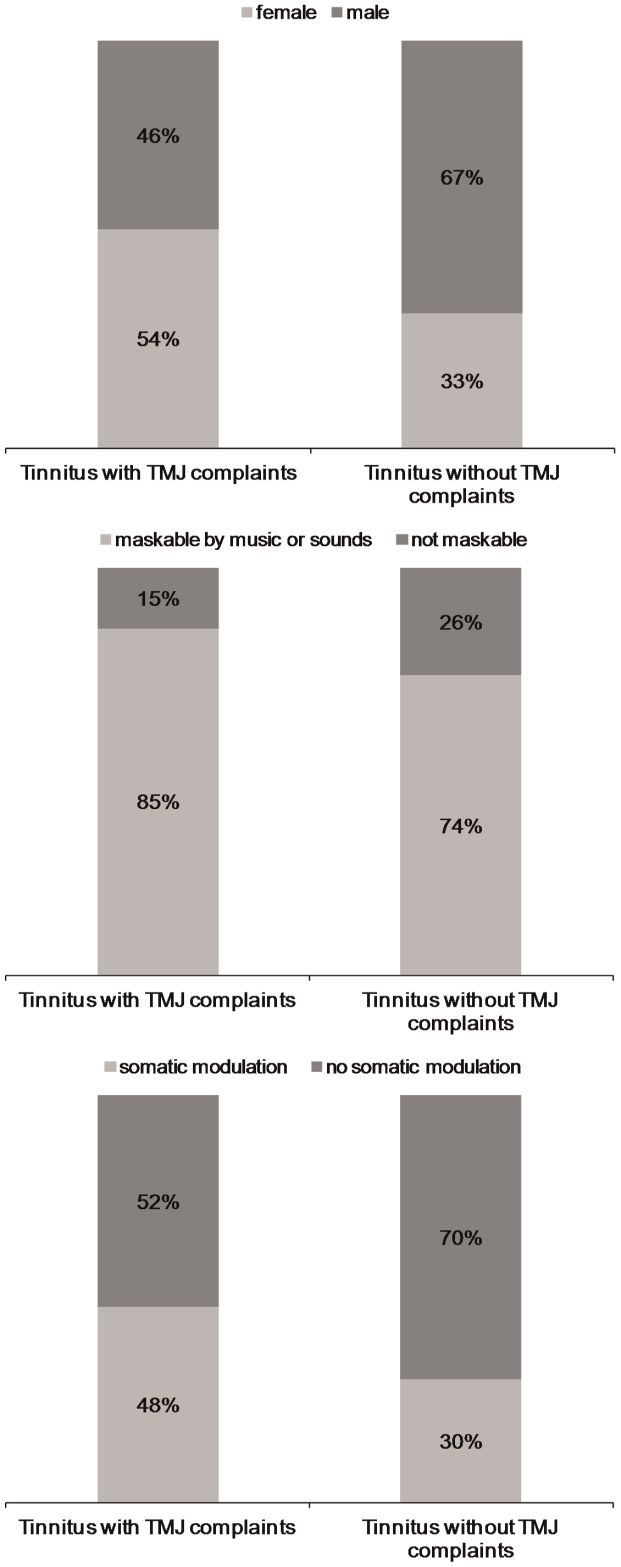
Relative proportion of gender, tinnitus maskability, and somatic modulation of tinnitus in dependence from complaints about temporomandibular joint disorder (categorical variables with significant contrasts between groups) (see separate documents).

Other tinnitus related aspects such as tinnitus duration, character of onset, pulsatile character, onset related events, hyperacusis, and hearing function did not show any significant differences between the two groups. In addition, scores of the THI, BDI and quality of life scales as well as numeric ratings with respect to tinnitus did not show group differences.

An overview of the results can be found in table 2. Figure 1 depicts results for gender, maskability of tinnitus with acoustic stimuli, and ability to modulate tinnitus by somatic maneuvers. Figure 1 depicts the relative frequencies of significant categorical variables as can be seen in table 2.

**Table 2:** Comparison of tinnitus patients with and without complaints about temporomandibular joint disorder (see separate documents).

Temporomandibular joint disorder complaints (n = 1204)	Yes (n = 261, 22%)		No (n = 943, 78%)	Statistics	
demographic characteristics					
age (years) (n = 1181)	50.7±13.9		53.6±13.1	T = 3.141; df = 1179; p = 0.002*	
age at tinnitus onset (years) (n = 1088)	41.5±14.5		45.2±14.4	T = 3.570; df = 1086; p<0.001*	
gender (female/male) (n = 1204)	140/121		311/632	χ^2^ = 37.245; df = 1; p<0.001*	
**tinnitus and audiologic characteristics**					
duration (n = 1089)	8.9±9.5		8.2±9.5	T = 0.947; df = 1087; p = 0.344	
onset (gradual/abrupt) (n = 1139)	114/136		442/447	χ^2^ = 1.325; df = 1; p = 0.250	
pulsatile (no/yes with heartbeat/yes other than heartbeat) (n = 1180)	196/34/27		758/90/75	χ^2^ = 4.486; df = 2; p = 0.106	
maskable by music or sounds (no/yes) (n = 1027)	33/190		213/591	χ^2^ = 13.107; df = 1; p<0.001*	
somatic modulation (no/yes) (n = 1183)	134/123		648/278	χ^2^ = 28.568; df = 1; p<0.001*	
onset related event (sound/whiplash/hearingloss/stress/headtrauma/others/multiple events/no event) (n = 1204)	17/11/25/61/5/62/44/36		54/12/95/219/6/253/154/150	χ^2^ = 14.556; df = 7; p = 0.042	
hyperacousis (never/rarely/some-times/usually/always) (n = 1181)	27/28/97/46/59		113/138/361/147/165	χ^2^ = 6.175; df = 4; p = 0.186	
mean hearing level (dB HL over all frequencies of both ears) (n = 849)	22.7±15.8		23.6±14.1	T = 0.767; df = 847; p = 0.443	
**questionnaires**					
tinnitus questionnaire (n = 981)	41.7±18.1		40.7±17.8	T = 0.709; df = 979; p = 0.479	
tinnitus handicap inventory (n = 1160)	50.4±22.2		47.2±23.3	T = 1.927; df = 1158; p = 0.054	
Beck depression inventory (n = 1117)	12.3±8.8		10.6±8.3	T = 2.722; df = 1115; p = 0.007	
WHO quality of life questionnaire domain 1 (n = 729)	13.9±3.2		14.5±2.9	T = 2.359; df = 727; p = 0.019	
WHO quality of life questionnaire domain 2 (n = 730)	13.7±2.8		14.1±2.7	T = 1.595; df = 728; p = 0.111	
WHO quality of life questionnaire domain 3 (n = 728)	14.2±3.5		14.5±3.1	T = 1.235; df = 726; p = 0.217	
WHO quality of life questionnaire domain 4 (n = 730)	15.4±2.7		15.9±2.3	T = 1.986; df = 728; p = 0.047	
**numeric rating scales (scale: 1–10)**					
loudness (n = 1138)	6.3±2.4		6.4±2.2	T = 0.682; df = 1136; p = 0.495	
discomfort (n = 1136)	7.0±2.3		7.0±2.4	T = 0.076; df = 1134; p = 0.939	
annoyance (n = 1139)	6.6±2.5		6.7±2.4	T = 0.806; df = 1137; p = 0.420	
ignorability (n = 1137)	6.6±2.8		6.8±2.7	T = 1.289; df = 1135; p = 0.198	
unpleasantness (n = 1140)	6.5±2.6		6.7±2.4	T = 1.110; df = 1138; p = 0.267	

* p<0.0022 (Bonferroni corrected significance threshold: 5% divided by 23 single contrasts).

## Discussion

In order to further substantiate the findings of a pilot study [12] we analyzed a large sample (1204 patients) from the TRI database to investigate the clinical characteristics of tinnitus patients with TMJ complaints in comparison to tinnitus patients without any TMJ complaints. In contrast to the former pilot study, in which the TMJ disorder was diagnosed by a specialized dentist, here information about TMJ complaints was obtained from patients’ self-report in the Tinnitus Case History Questionnaire [32]. We abstained from further differentiating the underlying pathology of the TMJ complaints, since the putative neurobiological mechanism for the interaction between TMJ complaints and tinnitus is abnormal trigeminal input to the dorsal cochlear nucleus [20], [21].

About one out of five tinnitus patients affirmed TMJ problems. Patients with comorbid TMJ complaints were more frequently female and of a younger age and had also experienced an earlier onset of tinnitus. All these findings are exactly in line with the results from the pilot study [12], suggesting that self-reported TMJ complaints are considered to be a reliable piece of information that proves diagnostic value in the assessment of tinnitus. However, we could not confirm a difference in hearing function between tinnitus patients with and without TMJ complaints. In the pilot study the difference in hearing function was driven primarily by patients with TMJ complaints as the primary complaint and such patients were not included in this study. Only patients presenting in a tinnitus clinic with the primary complaint of tinnitus were included in this study. Thus these findings further underscore that the sample recruitment strategy is of high relevance in the investigation of comorbidities of tinnitus [33].

In addition, we found that tinnitus patients with TMJ complaints can modulate their tinnitus by somatic maneuvers or by music and sound more frequently than tinnitus patients without TMJ problems. A possible association between TMJ complaints and somatic modulation has been postulated before [25], but to our knowledge our data are the first that empirically confirm this association. One earlier study with a substantial smaller sample size did not find such an association [14]. Since our study involved almost ten times more patients, this discrepancy may be related to study power. Whereas an association between TMJ comorbidity and the ability to modulate tinnitus by jaw, head or neck movements was expected, the significant difference in the rate of patients who could mask their tinnitus by environmental sounds, was an unexpected finding. Notably, this observation of higher masking rates in the tinnitus group with TMJ complaints is not confounded by hearing function, since there was no significant difference in audiometric data between the two groups. Rather this finding suggests that abnormal trigeminal input influences the interaction of tinnitus related neuronal abnormalities and the processing of auditory stimuli.

We are aware of the limitations of our study. First audiometric data were not available for the whole sample but only for 849 patients (66%). Moreover the criterion of TMJ complaints was based on self-report and we have no information about the exact underlying pathology and the laterality of the TMJ complaints. Thus further studies are needed to confirm our findings, to explore the relevance of the underlying TMJ pathology and to address the relation between TMJ laterality and tinnitus laterality.

Trigeminal somatosensoric input and auditory input converge at the dorsal cochlear nucleus (DCN) [34], [35]. This convergence at the DCN is generally considered to represent the neuronal correlate for the clinically observed interactions between the somatosensory system and tinnitus [16], [26]. Thus, one could speculate that abnormal auditory and trigeminal input to the DCN in patients with TMJ complaints leads to plastic changes of multisensoric processing in the DCN [25], [36], [37] which may provide an explanation for the observed higher rates of tinnitus modulation by both auditory and somatic modulation in this tinnitus subgroup. Support for this theory derives from recent animal experiments, which demonstrated plastic changes in the auditory-somatosensory integration in the DCN in noise-exposed animals, especially those that developed tinnitus [20]. Functional neuroimaging studies in tinnitus patients confirmed this interaction by demonstrating an enhanced response to jaw protrusion in cochlear nucleus and inferior colliculus in tinnitus patients as compared to controls [38].

The activation of the extralemniscal pathways has been proposed as an alternative explanation for the interaction between the somatosensory and the auditory system in tinnitus patients [23], [24]. This theory is based on the observation that electrical stimulation of the median nerve can modulate tinnitus loudness [39]. Finally TMJ disorders may influence tinnitus by modifying perceived hearing level at the middle ear. This theory could be further explored by investigating the relationship between the exact TMJ pathology and tinnitus.

The findings of this study are considered highly relevant in the quest for relevant clinical criteria for tinnitus subtyping, since it is clearly demonstrated that comorbid TMJ complaints exert an impact on the ability to modulate tinnitus by somatic maneuvers and sound. This difference in symptom modulation is likely to be relevant for the success of specific therapeutic interventions that involve auditory stimulation [40] or somatosensoric interventions [41]. Thus, based on our findings we propose TMJ complaints as a criterion for tinnitus subtyping and invite further studies to investigate its relevance in clinical practice.

In summary, our findings of reduced relevance of the risk factors “older age” and “male gender” together with higher rates of modulation by somatic or auditory stimuli in tinnitus patients with comorbid TMJ complaints suggest, that “comorbid TMJ complaints” represents a valuable criterion for defining a subgroup of tinnitus that exhibits clinical features that could be highly relevant, in future clinical research, for the evaluation of specific therapeutic interventions.

## References

[1] Langguth B, Kleinjung T, Landgrebe M (2011). Tinnitus: The Complexity of Standardization.. Eval Health Prof.

[2] Langguth B, Kleinjung T, Landgrebe M (2011). Severe tinnitus and depressive symptoms: a complex interaction.. Otolaryngol Head Neck Surg 145: 519; author reply 520.

[3] Langguth B, Landgrebe M, Kleinjung T, Sand GP, Hajak G (2011). Tinnitus and depression.. World J Biol Psychiatry.

[4] Chandra RK, Epstein VA, Fishman AJ (2009). Prevalence of depression and antidepressant use in an otolaryngology patient population.. Otolaryngol Head Neck Surg.

[5] Tyler R, Coelho C, Tao P, Ji H, Noble W (2008). Identifying tinnitus subgroups with cluster analysis.. Am J Audiol.

[6] Nam EC, Handzel O, Levine RA (2010). Carbamazepine responsive typewriter tinnitus from basilar invagination.. J Neurol Neurosurg Psychiatry.

[7] Costen JB (1997). A syndrome of ear and sinus symptoms dependent upon disturbed function of the temporomandibular joint. 1934.. Ann Otol Rhinol Laryngol.

[8] Wright EF, Bifano SL (1997). Tinnitus improvement through TMD therapy.. J Am Dent Assoc.

[9] Dolowitz DA, Ward JW, Fingerle CO, Smith CC (1964). The Role of Muscular Incoordination in the Pathogenesis of the Temporomandibular Joint Syndrome.. Laryngoscope.

[10] Chole RA, Parker WS (1992). Tinnitus and vertigo in patients with temporomandibular disorder.. Arch Otolaryngol Head Neck Surg.

[11] Bernhardt O, Mundt T, Welk A, Koppl N, Kocher T (2011). Signs and symptoms of temporomandibular disorders and the incidence of tinnitus.. J Oral Rehabil.

[12] Vielsmeier V, Kleinjung T, Strutz J, Burgers R, Kreuzer PM (2011). Tinnitus with Temporomandibular Joint Disorders: A Specific Entity of Tinnitus Patients?. Otolaryngol Head Neck Surg.

[13] Pinchoff RJ, Burkard RF, Salvi RJ, Coad ML, Lockwood AH (1998). Modulation of tinnitus by voluntary jaw movements.. Am J Otol.

[14] Sanchez TG, Guerra GC, Lorenzi MC, Brandao AL, Bento RF (2002). The influence of voluntary muscle contractions upon the onset and modulation of tinnitus.. Audiol Neurootol.

[15] Wright EF (2007). Otologic symptom improvement through TMD therapy.. Quintessence Int.

[16] Roberts LE, Eggermont JJ, Caspary DM, Shore SE, Melcher JR (2010). Ringing ears: the neuroscience of tinnitus.. J Neurosci.

[17] Dehmel S, Cui YL, Shore SE (2008). Cross-modal interactions of auditory and somatic inputs in the brainstem and midbrain and their imbalance in tinnitus and deafness.. Am J Audiol.

[18] Shore S, Zhou J, Koehler S (2007). Neural mechanisms underlying somatic tinnitus.. Prog Brain Res.

[19] Zhou J, Shore S (2004). Projections from the trigeminal nuclear complex to the cochlear nuclei: a retrograde and anterograde tracing study in the guinea pig.. J Neurosci Res.

[20] Dehmel S, Pradhan S, Koehler S, Bledsoe S, Shore S (2012). Noise overexposure alters long-term somatosensory-auditory processing in the dorsal cochlear nucleus–possible basis for tinnitus-related hyperactivity?. J Neurosci.

[21] Shore SE (2011). Plasticity of somatosensory inputs to the cochlear nucleus–implications for tinnitus.. Hear Res.

[22] Moller AR, Moller MB, Yokota M (1992). Some forms of tinnitus may involve the extralemniscal auditory pathway.. Laryngoscope.

[23] Moller AR (2003). Pathophysiology of tinnitus.. Otolaryngol Clin North Am 36: 249–266, v–vi.

[24] Moller AR (2007). The role of neural plasticity in tinnitus.. Prog Brain Res.

[25] Levine RA (1999). Somatic (craniocervical) tinnitus and the dorsal cochlear nucleus hypothesis.. Am J Otolaryngol.

[26] Levine RA, Nam EC, Oron Y, Melcher JR (2007). Evidence for a tinnitus subgroup responsive to somatosensory based treatment modalities.. Prog Brain Res.

[27] Latifpour DH, Grenner J, Sjodahl C (2009). The effect of a new treatment based on somatosensory stimulation in a group of patients with somatically related tinnitus.. Int Tinnitus J.

[28] Biesinger E, Reisshauer A, Mazurek B (2008). [The role of the cervical spine and the craniomandibular system in the pathogenesis of tinnitus. Somatosensory tinnitus].. HNO.

[29] Vanneste S, Plazier M, der Loo E, de Heyning PV, Congedo M (2010). The neural correlates of tinnitus-related distress.. Neuroimage.

[30] Kapoula Z, Yang Q, Le TT, Vernet M, Berbey N (2011). Medio-lateral postural instability in subjects with tinnitus.. Front Neurol.

[31] Landgrebe M, Zeman F, Koller M, Eberl Y, Mohr M (2010). The Tinnitus Research Initiative (TRI) database: a new approach for delineation of tinnitus subtypes and generation of predictors for treatment outcome.. BMC Med Inform Decis Mak.

[32] Langguth B, Goodey R, Azevedo A, Bjorne A, Cacace A (2007). Consensus for tinnitus patient assessment and treatment outcome measurement: Tinnitus Research Initiative meeting, Regensburg, July 2006.. Prog Brain Res.

[33] Langguth B, Landgrebe M, Kleinjung T, Sand GP, Hajak G (2011). Tinnitus and depression.. World J Biol Psychiatry.

[34] Shore SE (2011). Plasticity of somatosensory inputs to the cochlear nucleus - Implications for tinnitus.. Hear Res.

[35] Kaltenbach JA (2007). The dorsal cochlear nucleus as a contributor to tinnitus: mechanisms underlying the induction of hyperactivity.. Prog Brain Res.

[36] Kaltenbach JA (2006). Summary of evidence pointing to a role of the dorsal cochlear nucleus in the etiology of tinnitus.. Acta Otolaryngol.

[37] Tzounopoulos T (2008). Mechanisms of synaptic plasticity in the dorsal cochlear nucleus: plasticity-induced changes that could underlie tinnitus.. Am J Audiol.

[38] Lanting CP, de Kleine E, Eppinga RN, van Dijk P (2010). Neural correlates of human somatosensory integration in tinnitus.. Hear Res.

[39] Moller AR, Moller MB, Jannetta PJ, Jho HD (1992). Compound action potentials recorded from the exposed eighth nerve in patients with intractable tinnitus.. Laryngoscope.

[40] Vernon JA, Meikle MB (2003). Masking devices and alprazolam treatment for tinnitus.. Otolaryngol Clin North Am 36: 307–320, vii.

[41] Biesinger E, Kipman U, Schatz S, Langguth B (2010). Qigong for the treatment of tinnitus: a prospective randomized controlled study.. J Psychosom Res.

